# Supplementary material for: End Sequence Analysis Toolkit (ESAT) expands the extractable information from single-cell RNA-seq data
Source: Genome Res. 2016 Oct;26(10):1397–410. doi: 10.1101/gr.207902.116 (PMC5052061; doi:10.1101/gr.207902.116)
Supplement: Supplemental Material [file supp_gr.207902.116_Supp_Materials.tar.gz › ESAT-esat_v0.1/doc/html/functions_vars.html]

ESAT: Class Members - Variables


ESAT


- Main Page
- Related Pages
- Packages
- Classes
- Files

- Class List
- Class Index
- Class Hierarchy
- Class Members

- All
- Functions
- Variables
- Enumerator

- a
- b
- c
- d
- e
- f
- g
- h
- i
- k
- l
- m
- n
- o
- p
- q
- r
- s
- t
- u
- v
- w


All Classes Namespaces Files Functions Variables Enumerator Pages

### - a -

- A\_CODE
  : broad.core.multiplealignment.MultipleAlignment
- AcuI
  : broad.core.primer3.RestrictionEnzyme
- allNucleotides
  : broad.core.sequence.TranscribedSequenceComposition
- allSamples
  : broad.pda.seq.protection.MultiSampleScanPeakCaller
- ANALYSIS\_NAME
  : broad.pda.seq.rap.CollectAnnotationEnrichments
- ANNOTATION
  : broad.pda.seq.rap.GenomeCommandLineProgram
- annotation
  : umms.core.model.score.WindowScore.AbstractWindowScore
- ANNOTATION\_BASE
  : broad.pda.seq.rap.CollectAnnotationEnrichments
- ANNOTATION\_FILE
  : broad.pda.seq.rap.CountReads
  , broad.pda.seq.rap.PlotAggregateRegions
  , broad.pda.seq.rap.rna.CountReads
- ANNOTATIONS
  : broad.pda.seq.rap.CollectAnnotationEnrichments
- annotations
  : umms.core.annotation.AnnotationList< T extends Annotation >
- ARRAY\_140105
  : broad.pda.seq.rap.CountProbes


---

Generated on Fri Aug 15 2014 15:11:17 for ESAT by  

 1.8.7
